# Assessment of the Role of NO-cGMP Pathway in Orthodontic Tooth Movement Using PDE5 Inhibitors: An Animal Study

**Published:** 2016-11

**Authors:** Amir Hossein Mirhashemi, Mohammad Sadegh Ahmad Akhoundi, Rezvaneh Ghazanfari, Shahroo Etemad-Moghadam, Mojgan Alaeddini, Azam Khorshidian, Ahmad Reza Dehpour, Nafiseh Momeni

**Affiliations:** 1 Associate Professor, Dental Research Center, Dentistry Research Institute, Tehran University of Medical Sciences, Tehran, Iran; Department of Orthodontics, School of Dentistry, Tehran University of Medical Sciences, Tehran, Iran; 2 Professor, Laser Research Center, Dentistry Research Institute, Tehran University of Medical Sciences, Tehran, Iran; Department of Orthodontics, School of Dentistry, Tehran University of Medical Sciences, Tehran, Iran; 3 Assistant Professor, Department of Prosthodontics, School of Dentistry, International Campus, Tehran University of Medical Sciences, Tehran, Iran; 4 Associate Professor, Dental Research Center, Dentistry Research Institute, Tehran University of Medical Sciences, Tehran, Iran; 5 PhD Candidate, Dental Research Center, Dentistry Research Institute, Tehran University of Medical Sciences, Tehran, Iran; 6 Professor, Department of Pharmacology, Faculty of Medicine, Tehran University of Medical Sciences, Tehran, Iran

**Keywords:** Nitric Oxide, Phosphodiesterase 5 Inhibitors, Tooth Movement Techniques

## Abstract

**Objectives:**

Nitric oxide (NO) is a signaling molecule that mediates mechanical bone loading. Cyclic guanosine 3′, 5′ monophosphate (cGMP) is a NO-induced effector molecule. The aim of this study was to assess the effect of NO-cGMP pathway on orthodontic tooth movement (OTM) in rats by use of two phosphodiesterase 5 (PDE5) inhibitors namely sildenafil and tadalafil as chemical tools.

**Materials and Methods:**

Forty-five male Wistar rats were divided into three equal groups (n=15) based on the substance they received. The first group received daily injections of tadalafil; the second group received daily injections of sildenafil and the third group received daily injections of normal saline. The orthodontic appliances consisted of nickel-titanium closed-coil spring ligated between the maxillary right incisor and the first molar of the animals for 21 days. The amount of tooth movement was measured in all three groups at the end of this period. Histological analysis was performed to assess root resorption lacunae, osteoclast number and periodontal ligament (PDL) thickness.

**Results:**

All appliance-treated molars in the experimental and control groups showed evidence of tooth movement. The mean OTM was calculated to be 0.39±0.16, 0.32±0.16 and 0.26±0.16mm in tadalafil, sildenafil and control groups, respectively and there were no significant differences in OTM among the study groups (P>0.05). In the tadalafil group, significantly greater root resorption on the tension side was seen when compared with controls (P≤0.05).

**Conclusions:**

Tadalafil and sildenafil PDE-5 inhibitors affecting the NO-cGMP pathway did not affect OTM in rats.

## INTRODUCTION

Nitric oxide (NO) is involved in regulating bone turnover and bone cell function; it is a signaling molecule that mediates mechanical bone loading [1]. It has been shown that NO is involved in orthodontic tooth movement (OTM) as well [2–4]. The periodontal ligament (PDL) cells produce NO upon mechanical loading of teeth [5]. In many cells, the effects of NO are mediated by generation of guanosine 3′,5′ monophosphate (cGMP) [6]. But there is no consensus about the involvement of cGMP in metabolic pathways of mineralized tissue cells. Cyclic guanosine monophosphate is an intracellular regulator in both endocrine and non-endocrine mechanisms [7]. Evidence shows that NO affects osteoclastic bone resorption via a cGMP-independent mechanism [6]; but some have demonstrated that cGMP-dependent pathways may be involved in this process [8]. It has been reported that high concentrations of NO, such as those observed after stimulation with pro-inflammatory cytokines, have potent inhibitory effects on growth and differentiation of osteoblasts [8,9]. This may be due to the pro-apoptotic effects of NO on osteoblasts, and those effects are mediated partially through the cGMP pathway [8]. An initial study did not show a significant difference in level of cGMP when mechanical forces were applied to teeth [10] but a later study suggested that orthodontic forces may elevate NO production by PDL fibroblasts, which activates guanylyl cyclase in fibroblasts and leads to an increase in cGMP level [2]. Cyclic guanosine monophosphate is a cyclic nucleotide the synthesis of which is catalyzed via guanylate cyclase, and numerous cyclic nucleotide phosphodiesterases (PDE) can degrade it. Therefore, it is reasonable that the enhancement of cGMP level by inhibiting PDEs could effect on NO level and OTM; PDE5 inhibitors are used for this purpose. Eleven PDE subtypes have been identified; of which, PDE5 has been most extensively studied. It has been documented that PDE5 degrades 3′–5′-cGMP and its inhibition leads to an increase in intracellular cGMP levels and subsequent activation of protein kinase G, resulting in a decrease in Ca2+ influx and consequent relaxation of smooth muscles [11]. All tissues and cell types express PDE5; PDE5A1 and PDE5A2 are ubiquitous, but PDE5A3 is specific to smooth muscles [12,13]. Currently, little is known regarding the involvement of PDE5 in regulation of bone remodeling. Gong et al, [14] demonstrated that PDE5 inhibition may cause bone mass loss. They depicted schematic graphs showing a model for PDE5 inhibition regulation and bone homeostasis. Sildenafil and tadalafil are PDE-5 inhibitors shown to be effective in various pathological conditions via the NO pathway [15]. They differ in their selectivity, efficacy, side effects and pharmacokinetics. Preclinical trials have shown that tadalafil possesses an extended plasma half-life of 18 hours compared to three to four hours for sildenafil [16]. These two PDE-5 inhibitors prefer cGMP as a substrate. Tadalafil is an inhibitor of PDE-11 and inhibits the hydrolysis of both cAMP and cGMP [17]. We sought to assess the effect of NO-cGMP on OTM using PDE5 inhibitors as chemical tools.

## MATERIALS AND METHODS

The protocol of the current study was approved by the Ethics Committee of Tehran University of Medical Sciences (89-04-70-11825). The study was conducted in complete agreement with the Guide for the Care and Use of Laboratory Animals. A total of 45 male Wistar rats (200–250g) were housed in plastic cages, maintained on a 12/12-hour light/dark cycle and randomly divided into three groups (n=15). The first group received a daily intraperitoneal injection of tadalafil (10mg/kg), the second group received a daily intraperitoneal injection of sildenafil (10mg/kg) and the third group received a daily injection of normal saline. All rats were fed standard soft food in order to reduce the risk of appliance dislodging. The animals were weighed at the beginning of the study and right before sacrifice.

### Orthodontic treatment and measurement of tooth movement:

For orthodontic appliance placement, each rat was anaesthetized with intraperitoneal injection of xylazine HCL (6mg/kg) and ketamine (50mg/kg). The maxillary left molar was moved to the mesial space via ligation of 9mm, 0.010×0.030-inch nickel-titanium closed coil springs (Beijing Smart Technology Co., Beijing, China) with 0.010-inch stainless steel ligature wires between the molar and incisors. The technique of spring anchoring and force were similar to previous studies [18–22]. After 21 days, all animals were sacrificed by ether overdose. The mesial movement of the first molar was determined via measuring the space between the first and second molars by means of a feeler gauge before removal of the appliances to prevent distal relapse of the first molar.

All measurements were repeated twice by the same operator blinded to the study groups and the mean values were used for statistical analysis.

### Histological evaluation:

The maxillae were fixed in 10% formalin for five days and were placed in 5% formic acid until adequately decalcified (averagely five days). Routine processing was then performed and serial parasagittal sections (6μm thick) were prepared. The sections were stained with hematoxylin and eosin. The section containing the full length of the mesial root from the cementoenamel junction to the apex was selected and evaluated with a double-headed Olympus Bx-41 light microscope equipped with a digital camera (DP25 Olympus) and analysis software (DP2-BSW; Olympus, Tokyo, Japan). Two observers analyzed the number of osteoclasts, PDL width, the number of resorption lacunae and their maximum depth and width and coronal and apical widths of the PDL on both mesial and distal aspects of the mesial root. All sections were measured twice by both observers under the double-headed microscope and the mean of the two values was used in all consecutive calculations.

### Statistical analysis:

Data were subjected to one-way ANOVA. To understand the significance of differences, Tukey’s test as a post hoc analysis was applied. P-value less than 0.05 was considered statistically significant.

## RESULTS

At the end of 21 days of orthodontic treatment in rats, there were no significant differences in overall weight among the study groups. All appliance-treated molars drifted towards the incisors (Table 1); but significant differences were not found in amounts of tooth movement among the groups (P=0.14). The highest and the lowest amounts of OTM were observed in the tadalafil and control groups, respectively. Table 2 shows descriptive histological data. The number, depth and width of distal resorption lacunae revealed significant differences among the study groups, and Tukey’s test exhibited differences between the tadalafil and control groups in all three parameters. There were no statistically significant differences in the number of osteoclasts (P=0.6) or in the widths of the PDL in the mesio-apical (P=0.8), disto-apical (P=0.1), mesio-coronal (P=0.1) and disto-coronal (P=0.2) areas of the mesio-buccal molar root.

**Table 1:** Molar separation mean in millimeters during a 21-day period (n=15)

** Group **	** Molar separation mean (mm) **	** SD **	** SE **	** 95% CI **		** Minimum **	** Maximum **

				Lower	Upper		
** Tadalafil **	0.39	0.16	0.04	0.30	0.49	0.10	0.65
** Sildenafil **	0.32	0.16	0.04	0.22	0.42	0.15	0.55
** Control **	0.26	0.16	0.04	0.16	0.37	0.11	0.60

SD: Standard deviation; SE: Standard error; CI: Confidence interval

**Table 2:** Comparison of mesiobuccal root resorption after 21 days of load application

	** Mean (SD) number of RL **		** Mean (SD) RL width (micrometers) **		** Mean (SD) RL depth (micrometers) **	

	** Compression **	** Tension **	** Compression **	** Tension **	** Compression **	** Tension **
** Tadalafil **	2.0(1.4)	2.1(1.8)	62.5(57.2)	84.1(51.8)	30.8(31.5)	42.4(28.9)
** Sildenafil **	2.5(1.9)	1.7(1.5)	52.0(45.3)	65.6(87.4)	23.6(10.7)	29.5(30.2)
** Control **	1.8(1.6)	0.6(1.3)	28.2(22.5)	17.4(36.2)	14.5(9.3)	9.2(19.2)
** P-value **	0.50	0.04	0.13	0.02	0.12	0.00

RL: Resorption lacunae; SD: Standard deviation

## DISCUSSION

The study results revealed that the mean OTM was not significantly different in the control, tadalafil and sildenafil groups.

Gong et al, [14] reported that two-month treatment with PDE5 inhibitors at a high dosage (45 or 75mg/kg) may potentially cause bone catabolism; they investigated osteoblastogenesis by bone marrow-derived stromal cells in rats and observed reduction of bone mass. Since the dosage and duration of drug that they administrated were higher than those in our study, our results were different from theirs.

Shirazi et al, [2] stated that orthodontic forces may elevate NO production by the PDL fibroblasts and increase bone remodeling and OTM. They stated that NO activates guanylate cyclase in the PDL and causes a rise in the level of cGMP. In addition, Yaroslavskiy et al, [23] declared that NO or cGMP regulate the function of osteoclasts and bone resorption. Ralston and Grabowski [6] also examined the role of NO and cGMP in bone resorption and showed that cytokine-induced NO production was accompanied by increased production of cGMP; but they concluded that it had no significant effect on bone resorption, and the effects of NO on bone resorption were not mediated through the cGMP pathway. Stanfeld et al, [10] studied the biochemical aspects of OTM and reported that there was no significant difference in cGMP levels between treated and control sites when orthodontic forces were applied to cat’s teeth. As mentioned earlier, administration of PDE5 inhibitors leads to an increase in level of cGMP; therefore, the present outcomes could confirm the results of studies that reported cGMP had no significant effect on bone resorption and OTM. Further studies on various molecular features of OTM are necessary to evaluate the role of NO-cGMP pathway in OTM.

It has been reported that cAMP is the second messenger system classically associated with mechanical force transduction [24], and its elevation induces osteoclast formation [25]. Tadalafil has shown PDE-11 selectivity [16]. PDE-11 is a dual-substrate PDE, which hydrolyzes both cAMP and cGMP [26]. The enhancement of OTM in samples that received tadalafil was probably due to cAMP increase.

The histomorphometric findings in our study did not show any significant difference in osteoclast number among the groups. This supports our OTM data and is in agreement with the findings of Takami et al, [27] who indicated that PDE-5 inhibitors did not induce osteoclast formation in the calvaria. Histing et al, [28] reported that sildenafil did not significantly affect the balance between receptor activator of nuclear factor κ B ligand/receptor activator of nuclear factor κ B and osteoprotegerin.

Our results revealed that in the tadalafil group, the number, depth and width of resorption lacunae at the tension side (distal aspect of molar root) were significantly greater than those of resorption lacunae at the same site in controls. This could be attributed to the effects of vascular endothelial growth factors (VEGFs) produced by the mechanically activated fibroblasts at the tension side. They are the primary mediators of angiogenesis and increase vascular permeability [29]. The VEGFs were found in large cells that appeared in clusters around the root resorption lacunae. Also, osteoclasts lining the bone around the lesion were VEGF positive [30]. Presence of VEGFs on osteoclasts indicates that the resorption activity is influenced by them [31]. It has been documented that sildenafil and tadalafil induce VEGFs [32].

In the current study, administration of two types of PDE-5 inhibitors (tadalafil and sildenafil), which enhance cGMP, had no significant effect on OTM. However, our experiment was conducted on a small laboratory animal and thus, the results cannot be extrapolated to humans.

## CONCLUSION

Tadalafil and sildenafil as PDE-5 inhibitors administrated to evaluate the role of NO-cGMP pathway in OTM did not interfere with bone remodeling or OTM in rats.
